# 0095. Transvenous vagus nerve stimulation does not modulate the innate immune response in humans *in vivo* during experimental endotoxemia

**DOI:** 10.1186/2197-425X-2-S1-P7

**Published:** 2014-09-26

**Authors:** M Kox, LT van Eijk, T Frenzel, T Verhaak, JF Gerretsen, JG van der Hoeven, L Kornet, A Scheiner, P Pickkers

**Affiliations:** Radboud University Medical Center, Intensive Care Medicine, Nijmegen, Netherlands; Radboud Institute for Infectious Diseases, Nijmegen, Netherlands; Medtronic Inc., Maastricht, Netherlands; Medtronic Inc., St. Paul, MN USA

## Introduction

In a variety of conditions excessive and/or persistent activation of the innate immune system has detrimental effects. In animals, electrical vagus nerve stimulation (VNS) inhibits the innate immune response in models of endotoxemia (administration of lipopolysaccharide [LPS]), sepsis, trauma, and hemorrhagic shock, via the so-called cholinergic anti-inflammatory pathway. However, human *in vivo* evidence is lacking. Up till now, VNS was possible through implantation of a cuff electrode wrapped around the nerve, which limits its use in acute inflammatory situations frequently encountered on the ICU. A novel, less invasive VNS method is transvenous VNS (tVNS).

## Objective

To determine whether tVNS exerts anti-inflammatory effects during experimental human endotoxemia.

## Methods

A parallel randomized double-blind sham-controlled study in healthy male volunteers was performed. Subjects were randomized to receive either tVNS (n=10) or sham tVNS (n=10).

In both groups, a stimulation catheter with multiple circular electrode pairs was inserted in the left internal jugular vein at C5-C7 spinal level to be situated adjacent to the vagal nerve. In the tVNS group, stimulation (0-10 V, 1 ms, 20 Hz) was continuously performed during 30 minutes, starting 10 minutes before intravenous administration of 2 ng/kg *E. Coli* LPS. In sham subjects, the exact same procedures were performed, but the stimulator was not switched on by an unblinded team member.

## Results

**Figure 1:**
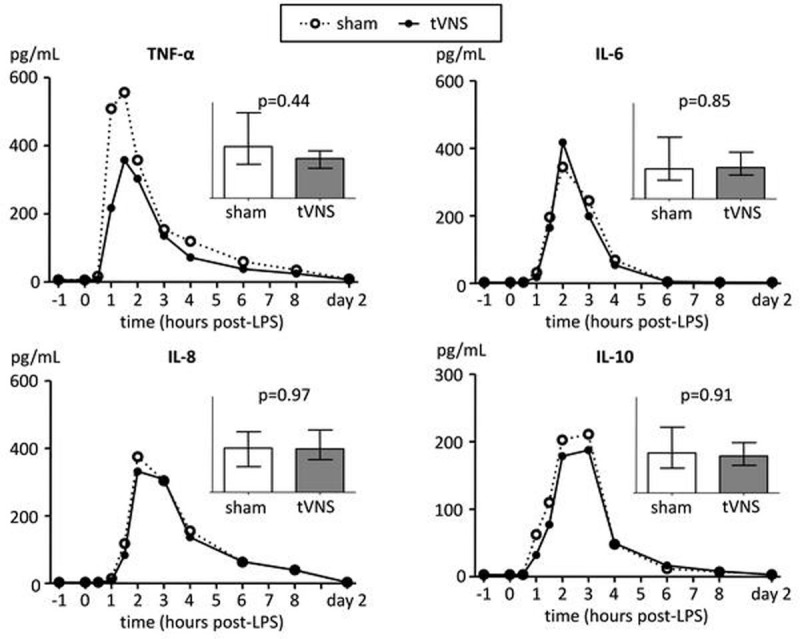
Plasma concentrations of inflammatory cytokines during endotoxemia. Data are expressed as medians od 10 subjects per group. Inserted bar graphs depict median interquartile range of area under curve (AUC) of cytokines. P values calculated using Mann-Whitney U-tests.

**Figure 2:**
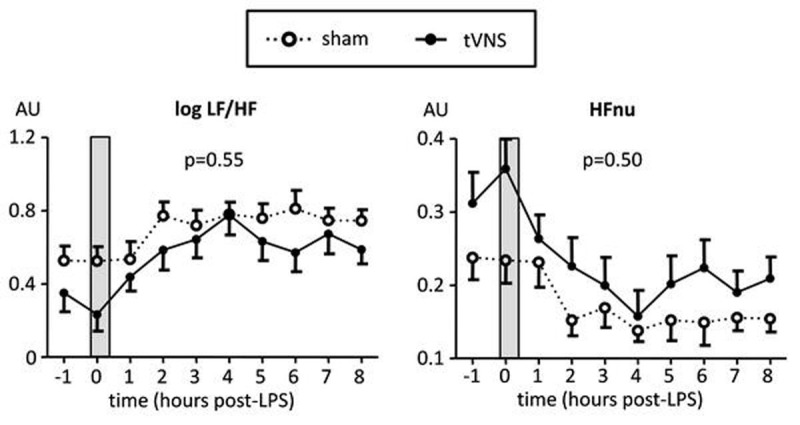
Heart rate variability parameters during endotoxemia. Left panel: ratio between low frequency and high frequency power (LF/HF). Right panel: high frequency power in normalized units (HFnu). Data are expressed as mea SEM of 10 subjects per group. LH/HF was log-transformed to attain normal distribution. Gray box indicates period in which the (sham) stimulation took place. P-values indicate differences between groups over time calculated using repeated measures two-way analysis of variance (ANOVA, interaction term). AU: arbitrary units.

In all 20 subjects placement of the stimulation electrode was successful and uneventful. Furthermore, in all subjects of the tVNS group, laryngeal vibration was confirmed by an unblinded team member, indicating stimulation of vagal fibers. LPS administration resulted in an increase in heart rate of 26±3 bpm and a decrease in mean arterial pressure of 16±2 mmHg, as well as fever (increase of 1.5±0.1 ^o^C) and flu-like symptoms. No differences between groups were observed. Furthermore, plasma levels of inflammatory cytokines increased sharply, but responses were similar between groups (Figure 1). Likewise, cytokine production by leukocytes *ex vivo* restimulated with LPS as well as neutrophil phagocytosis capacity were unaffected by tVNS. Finally, heart rate variability analysis revealed that during (sham) stimulation, the ratio between low frequency and high frequency (HF) spectral power was significantly lower, and HF power in normalized units significantly higher in the tVNS group compared with the sham group, suggestive of increased parasympathethic activity (Figure 2, p=0.02 for both indices, unpaired Student's t-test at T=0). However, when corrected for baseline differences, this effect was no longer statistically significant (p=0.35 for both indices).

## Conclusions

Thirty-minute transvenous vagus nerve stimulation is feasible and safe but does not modulate the innate immune response in humans in vivo during experimental human endotoxemia.

